# Securing the Insecure: A First-Line-of-Defense for Body-Centric Nanoscale Communication Systems Operating in THz Band

**DOI:** 10.3390/s21103534

**Published:** 2021-05-19

**Authors:** Waqas Aman, Muhammad Mahboob Ur Rahman, Hasan T. Abbas, Muhammad Arslan Khalid, Muhammad A. Imran, Akram Alomainy, Qammer H. Abbasi

**Affiliations:** 1 Electrical Engineering Department, Information Technology University, Lahore 54000, Pakistan; waqas.aman@itu.edu.pk (W.A.); mahboob.rahman@itu.edu.pk (M.M.U.R.); 2 Department of Electronics and Nano Engineering, University of Glasgow, Glasgow G12 8QQ, UK; Hasan.Abbas@glasgow.ac.uk (H.T.A.); Muhammad.Imran@glasgow.ac.uk (M.A.I.); 3 Division of Biomedical Engineering, School of Engineering, University of Glasgow, Glasgow G12 8QQ, UK; arslan.k@live.com; 4 Artificial Intelligence Research Center (AIRC), Ajman University, Ajman 00000, United Arab Emirates; 5 School of Electronic Engineering and Computer Science, Queen Mary University of London, London E1 4NS, UK; a.alomainy@qmul.ac.uk

**Keywords:** body-centric sensor networks, nanoscale communication, terahertz communication, nano sensors, security, authentication, outlier detection, sensor networks, healthcare systems

## Abstract

This manuscript presents a novel mechanism (at the physical layer) for authentication and transmitter identification in a body-centric nanoscale communication system operating in the terahertz (THz) band. The unique characteristics of the propagation medium in the THz band renders the existing techniques (say for impersonation detection in cellular networks) not applicable. In this work, we considered a body-centric network with multiple on-body nano-senor nodes (of which some nano-sensors have been compromised) who communicate their sensed data to a nearby gateway node. We proposed to protect the transmissions on the link between the legitimate nano-sensor nodes and the gateway by exploiting the path loss of the THz propagation medium as the fingerprint/feature of the sender node to carry out authentication at the gateway. Specifically, we proposed a two-step hypothesis testing mechanism at the gateway to counter the impersonation (false data injection) attacks by malicious nano-sensors. To this end, we computed the path loss of the THz link under consideration using the high-resolution transmission molecular absorption (HITRAN) database. Furthermore, to refine the outcome of the two-step hypothesis testing device, we modeled the impersonation attack detection problem as a hidden Markov model (HMM), which was then solved by the classical Viterbi algorithm. As a bye-product of the authentication problem, we performed transmitter identification (when the two-step hypothesis testing device decides no impersonation) using (i) the maximum likelihood (ML) method and (ii) the Gaussian mixture model (GMM), whose parameters are learned via the expectation–maximization algorithm. Our simulation results showed that the two error probabilities (missed detection and false alarm) were decreasing functions of the signal-to-noise ratio (SNR). Specifically, at an SNR of 10 dB with a pre-specified false alarm rate of 0.2, the probability of correct detection was almost one. We further noticed that the HMM method outperformed the two-step hypothesis testing method at low SNRs (e.g., a 10% increase in accuracy was recorded at SNR = −5 dB), as expected. Finally, it was observed that the GMM method was useful when the ground truths (the true path loss values for all the legitimate THz links) were noisy.

## 1. Introduction

Nanoscale communication systems have attracted researchers due to their promising applications in healthcare, manufacturing industries, environmental control, etc. [1]. On the other hand, body-centric communication has potential applications in healthcare, entertainment, etc. [2]. Generally, body-centric communication is classified as “off”-, “on”-, and “in”-body communication based on the communication among implanted or wearable electronic devices. In this work, we focused on the body-centric communication systems where nano sensors/devices operating in the THz band are deployed on the body of a human being.

Due to the small size of nano devices, the existing frameworks, techniques, and methods proposed for communication networks such as WiFi, 4G, etc., are not suitable for exchanging information amongst the nano devices [3]. For instance, nano devices are unable to operate at microwave bands due to their small size. They would require molecular communication and the terahertz (THz) band for operation. Additionally, in IoT devices, due to the small energy sources, the computational processing capability is limited. Therefore, it is necessary to meet the requirements for new protocols of nano devices at all layers of the protocol stack. Operating in the THz band (0.1–10 THz) is a promising solution at the physical layer (PL) [4], which makes the antenna size very small and thus suitable for exchanging information between nano devices.

**Figure 1:**
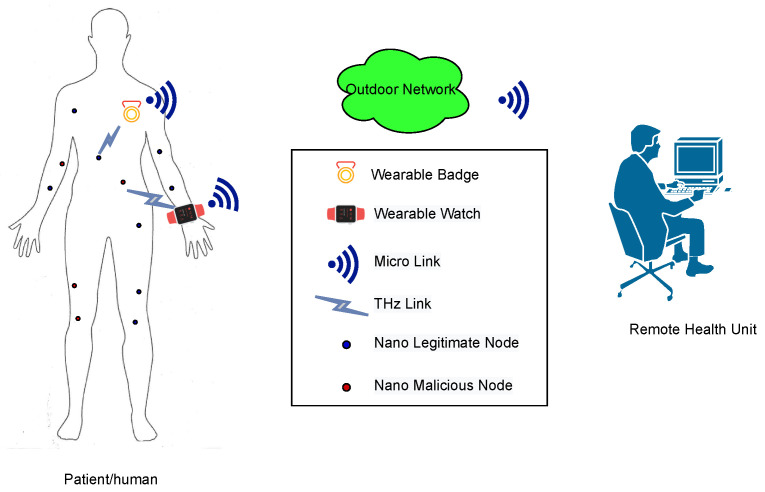
An envisioned future body-centric nanoscale healthcare system with possible malicious nodes.

Like other communication networks, the body-centric nanoscale communication networks are also prone to a wide range of active and passive attacks by adversaries [5]. Some of the common attacks include eavesdropping, impersonation, denial of service (DoS), etc. Here, we investigated an impersonation attack in body-centric nanoscale communication networks. Figure 1 shows an illustration of an impersonation attack on a smart healthcare system scenario. The nano nodes are deployed on the body of a person/patient for disease diagnostics or to remotely monitor his/her health parameters. These nano devices are connected to a wearable device, which communicates the data to an outdoor network via a nano-to-micro interface. Assuming an enemy of the person secretly deployed its own nano machines nearby with the aim of impersonating the person’s legitimate nodes to report false measurements to the remote health unit, an incorrect response through the nano machines or nearby doctors could result in devastating consequences. Therefore, we need an authentication mechanism at the nano-to-micro interface device (wearable device) to allow data transmission (reported measurements, i.e., glucose, blood pressure, etc., of nano nodes/sensors) from legitimate nano nodes only, blocking all malicious nodes.

In traditional communication systems, the countermeasures for such attacks are performed at the higher layer using cryptography. Despite the wide work in the field of cryptography, the mechanism can be compromised because of its sole dependency on the predefined shared secret among the legitimate users. With recent advances in quantum computing, traditional encryption has become vulnerable to being easily decoded, and existing crypto-based measures are not quantum secure unless the size of secret keys increases to impractical lengths [6]. In this regard, physical layer (PL) security finds itself as a promising mechanism in future communication systems. PL security exploits the random nature of the physical medium/layer for security purposes [7].

Authentication is one of the pillars required for the security of any communication system. PL authentication is a systematic procedure that uses PL’s features to provide authentication. In conventional systems, asymmetric key encryption (AKE) is typically used in the authentication phase, which is the realm of public key encryption (a crypto-based approach). Such schemes are quantum insecure and incur overhead or high computations, which not only increase the size of the device, but also consume much power. The devices fabricated for nanoscale communication are energy constrained as they incorporate a small source of energy (a battery). PL authentication has a low overhead (a simple procedure that typically includes feature estimation and testing) and is almost impossible to clone unless the devices lie on each other. Various fingerprints including RSS [8], CIR [9,10], CFR [11,12], carrier frequency offset [13], and I/Q imbalance [14] have been reported for PL authentication in conventional communication systems.

Related Work: The authors in [15] for the first time studied authentication using path loss (S21 parameter) in body-centric communication using millimeter waves. Regarding the security of systems operating in the THz band, we found some works [5,16,17,18] in the literature. The work [16] provided the first study on the security challenges faced by nanoscale communication systems, while the work [17] presented some possible promising applications along with the security challenges in the Internet of Nano-Things. Further, the experimental work of Jianjun et al. [5] for the first time rejected the claim about security in the THz band. The claim was that the inherit narrow beamwidth of the THz link makes it secure and thus impossible for a malicious node to accomplish an eavesdropping attack. The authors in [5] in their experiments used reflectors of different shapes between the THz transmitter and receiver. Then, with the help of secrecy capacity and blockage as performance metrics, they clearly demonstrated that eavesdropping attacks in the THz band can be easily performed.

The differences between our work and previous work are as follows: The first work [15], which studied the authentication problem in body-centric communication systems, considered millimeter-wave communication with a three-node setup. In contrast, our work considered multiple legitimate and malicious nodes operating in the THz band. The work [5] considered an eavesdropping attack in a system operating in the THz band, which was a different problem/attack than the attack we considered in our work. Next, in our previous work [18], we studied PL authentication for an in vivo nanoscale communication system whereby we utilized the path loss as the device fingerprint for a three-node system (i.e., Alice, Eve, and Bob). The difference between our previous work [18] and this work was twofold. First, the previous work was limited to the three-node system only, while in this work, the system model was comprised of multiple legitimate and malicious nodes. Second, the previous work was for an in vivo nanoscale communication system where authentication occurs at a nano node (Bob).

Contributions: For the first time, this work studied authentication at a nano-to-micro interface device (wearable device) in an on-body-centric communication system where we exploited the high-resolution transmission molecular absorption (HITRAN) database [19] for computing the path loss. For the first time, impersonation attack detection at the wearable device/receiver/Bob in multiple legitimate and malicious nano nodes operating in the THz band is performed via different mechanisms. We performed authentication by two-step hypothesis testing. We refined the output of the hypothesis testing via the hidden Markov model (HMM) with the Viterbi algorithm. We also performed transmitter identification via the maximum likelihood and Gaussian mixture model (GMM) with the expectation–maximization algorithm.

Outline: The rest of this paper is organized as follows. Section 2 provides the system model. Section 3 discusses authentication via two-step hypothesis testing. Section 4 presents the hidden Markov model to refine the output of hypothesis testing. Section 5 provides transmitter identification schemes. Section 6 presents simulation results with discussions, and Section 7 concludes the paper.

## 2. System Model

**Figure 2:**
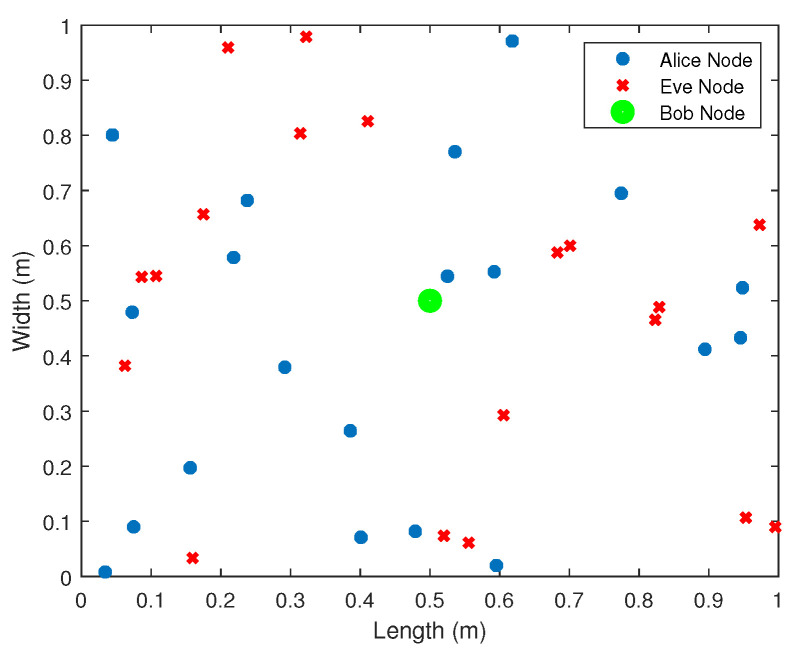
System model for the simulation purposes: Bob is placed at the origin. Alice’s and Eve’s node locations are modeled as uniformly distributed random variables. In this case, M=10 and N=10.

For the purposes of the simulation, we considered a square 2D map/layout of size (1 m × 1 m) where M+N nano transmission (Tx) nodes, *M* Alice (legitimate) nodes ${\left\{A_{i}\right\}}_{i=1}^{M}$, and *N* Eve (malicious) nodes ${\left\{E_{j}\right\}}_{j=1}^{N}$ are deployed according to the uniform distribution model, whilst a nano-to-micro interface device/receiver node, Bob, is placed at the origin, as shown in Figure 2. We assumed that the Tx nodes transmitted with a fixed/pre-specified transmit power so that the path loss can be computed by Bob.

The path loss is given as [20,21]:

(1) $$\begin{array}{c} L\left(f,d\right)\left[dB\right]=L_{a}\left(f,d\right)\left[dB\right]+L_{s}\left(f,d\right)\left[dB\right], \end{array}$$

where *f* is the frequency, *d* is the distance, $L_{a}\left(f,d\right)\left[dB\right]$ is the absorption loss, and $L_{s}\left(f,d\right)\left[dB\right]$ is the spreading loss. More details of spreading and absorption losses are given in Appendix A.

In the next section, we discuss the two-step mechanism for impersonation detection.

## 3. Authentication via Two-Step Hypothesis Testing

We assumed that the shared channel is time-slotted, whilst the transmit nodes perform channel sensing before transmitting; hence, there are no collisions. Without loss of generality, it can be assumed that $A_{i}$ is the legitimate node for slot *k*, but if $A_{i}$ does not transmit during this time slot, $E_{j}$ could transmit to Bob pretending to be an Alice node. Therefore, Bob needs to authenticate each message received on the shared channel and verify the transmitter identity (if no impersonation has been declared) in a systematic manner.

Assume that the noisy measurement z(k)=L+n(k) has been obtained at time *k* (for instance, by using the pulse-based method as discussed in [22]), where $n\left(k\right)∼N\left(0,\sigma^{2}\right)$ and *L* is the path loss. Furthermore, in line with previous studies [18,23], we assumed that Bob has already learned the ground truth via prior training on a secure channel. The ground truth vector can be denoted by $l={\left\{L_{1},...,L_{M}\right\}}^{T}$. The two-step hypothesis testing or maximum likelihood (ML) hypothesis test can be explained by the following equations:

(2) $$\left(T^{*},i^{*}\right)=\min_{i}\;\left|z-L_{i}\right|.$$

Next, the binary hypothesis test works as follows:

(3) $$\left\{\begin{array}{cc} H_{0}\left(\mathrm{no}\;\mathrm{impersonation}\right): & T^{*}=\min_{i}\left|z\left(k\right)-L_{i}\right|<\epsilon \\ H_{1}\left(\mathrm{impersonation}\right): & T^{*}=\min_{i}\left|z\left(k\right)-L_{i}\right|>\epsilon \end{array}\right..$$

Equivalently, we have:

(4) $$\begin{array}{c} T^{*}{≷}_{H_{0}}^{H_{1}}\epsilon , \end{array}$$

where ϵ is a small threshold—a design parameter. This work followed the Neyman–Pearson theorem [24], which states that, for a pre-specified $P_{fa}$, ϵ can be chosen such that $P_{md}$ is minimized.

The error probabilities for the above hypothesis tests are:

(5) $$\begin{array}{cc} P_{fa} & =P\left(H_{1}|H_{0}\right)=\sum_{i=1}^{M}P\left(T^{*}>\epsilon |A_{i}\right)\pi \left(i\right)\\ \; & =\sum_{i=1}^{M}2Q\left(\frac{\epsilon }{\sigma }\right)\pi \left(i\right)=2Q\left(\frac{\epsilon }{\sigma }\right)\sum_{i=1}^{M}\pi \left(i\right)=2Q\left(\frac{\epsilon }{\sigma }\right), \end{array}$$

where $Q\left(x\right)=\frac{1}{\sqrt{2}\pi }\int_{x}^{\infty }e^{\frac{-t^{2}}{2}}dt$ is the complementary cumulative distribution function (ccdf) of a standard normal distribution, and π(i) is the prior probability of $A_{i}$. Thus, the threshold could be computed as follows:

(6) $$\epsilon =\sigma Q^{-1}\left(\frac{P_{fa}}{2}\right).$$

Then, $P_{md}$ is given as:

(7) $$\begin{array}{cc} \; & P_{md}=P\left(H_{0}|H_{1}\right)=P\left(T^{*}<\epsilon |H_{1}\right)\\ \; & =\sum_{j=1}^{N}\sum_{i=1}^{M}\left[Q\left(\frac{L_{i}-L_{j}-\epsilon }{\sigma }\right)-Q\left(\frac{L_{i}-L_{j}+\epsilon }{\sigma }\right)\right]\pi \left(j\right), \end{array}$$

where $\pi \left(j\right)=\sum_{i=1}^{M}\alpha_{ij}\pi \left(i\right)$ is the prior probability of $E_{j}$. $0<\alpha_{ij}<1$ is the fraction of slots that were originally dedicated to $A_{i}$, but were found idle and thus utilized by $E_{j}$.

Since $P_{md}$ is an R.V., the expected value ${\bar{P}}_{md}:=E\left(P_{md}\right)$ is as follows:

(8) $$\begin{array}{cc} \; & {\bar{P}}_{md}=\sum_{j=1}^{N}\frac{1}{\Delta }\pi \left(j\right)\\ \; & \left(\int_{L_{min}}^{L_{max}}\sum_{i=1}^{M}Q\left(\frac{L_{i}-L_{j}^{\left(E\right)}-\epsilon }{\sigma }\right)-Q\left(\frac{L_{i}-L_{j}^{\left(E\right)}+\epsilon }{\sigma }\right)dL_{j}^{\left(E\right)}\right)\\ \; & =\sum_{j=1}^{N}\frac{1}{\Delta }\pi \left(j\right).\\ \; & \left(\int_{L_{min}}^{L_{max}}\sum_{i=1}^{M}Q\left(\frac{L_{i}-L^{\left(E\right)}-\epsilon }{\sigma }\right)-Q\left(\frac{L_{i}-L^{\left(E\right)}+\epsilon }{\sigma }\right)dL^{\left(E\right)}\right), \end{array}$$

where we assumed that the unknown path loss $L_{j}∼U\left(L_{min},L_{max}\right)$∀j and $\Delta =L_{max}-L_{min}$.

Next, we discuss the HMM for refining the outcomes/results of the two-step hypothesis testing.

## 4. Hidden Markov Model-Based Approach

To refine the output of the two-step hypothesis testing, we used the HMM-based approach. More specifically, at a given time instant *k*, the system is in one of the two states with the state-space: $S=\{s_{0},s_{1}\}$. The states $s_{0}$ and $s_{1}$ imply that there is no impersonation, impersonation respectively, at time *k*. However, the true state of the system is hidden; therefore, what we observe through the hypothesis test is another observable Markov chain. The connection between the true/hidden state and the observable state is given by the emission probability matrix:

(9) $$R=\left[\begin{array}{cc} r_{0,0} & r_{0,1}\\ r_{1,0} & r_{1,1} \end{array}\right],$$

where $r_{i,j}=Pr\left(x\left[k\right]=i|s\left[k\right]=j\right)$, i,j∈{0,1}. The off-diagonal elements in the *i*-th row of R represents the errors made by the ML test, i.e., deciding the state as s[k]=j, j∈{0,1}∖i while the system was actually in state s[k]=i.

The transition from state *i* to state *j* occurs after a fixed interval of $T=t_{k}-t_{k-1}$ seconds where 1/T is the measurement rate. Assume that the system was in state $s_{0}$ at time k=0, i.e., $x\left[0\right]={\left[1,0\right]}^{T}$, we are in time k−1 and want to predict the probability vector x[k] at time *k*, and the system is in state $s_{i}$, i∈{0,1}. To this end, we have the following transition probability matrix:

(10) $$P=\left[\begin{array}{cc} p_{0,0} & p_{0,1}\\ p_{1,0} & p_{1,1} \end{array}\right],$$

where $p_{i,j}=P\left(x\left[k\right]=j|x\left[k-1\right]=i\right)$, i,j∈{0,1}. Then, we have the following relation: $x\left[k\right]=P^{k}x\left[0\right]$. Alternatively, we can write: x[k]=Px[k−1].

### ML Estimation of a Hidden Markov Sequence Using the Viterbi Algorithm

The Viterbi algorithm is used for the ML sequence estimation (MLSE) of ${\left\{s\left[k\right]\right\}}_{k=1}^{K}$, given ${\left\{x\left[k\right]\right\}}_{k=1}^{K}$ as:

(11) $$\left\{s\left[k\right]\right\}=\arg\max_{\left\{s^{{}^{\prime }}\left[k\right]\right\}}p\left(x\left[k\right]|s^{{}^{\prime }}\left[k\right]\right).$$

At this stage, we are done with impersonation detection mechanisms. Next, we discuss the transmitter identification mechanisms.

## 5. Transmitter Identification

The transmitter identification is accomplished via two approaches: ML- and GMM-based transmitter identification.

### 5.1. ML-Based Approach

In the ML-based approach, the probability of the misclassification error resulting from Equation (2) is given as:

(12) $$P_{mc}=\sum_{i=1}^{M}P_{mc|i}\;\pi \left(i\right),$$

where $P_{mc|i}=P\left(\mathrm{Bob}\;\mathrm{decides}\;A_{j}|A_{i}\;\mathrm{was}\;\mathrm{the}\;\mathrm{sender}\right)$. For the hypothesis test of (4), $P_{mc|i}$ is given as:

(13) $$P_{mc|i}=1-\left(Q\left(\frac{{\tilde{L}}_{l,i}-{\tilde{L}}_{i}}{\sigma }\right)-Q\left(\frac{{\tilde{L}}_{u,i}-{\tilde{L}}_{i}}{\sigma }\right)\right),$$

where ${\tilde{L}}_{l,i}=\frac{{\tilde{L}}_{i-1}+{\tilde{L}}_{i}}{2}$, ${\tilde{L}}_{u,i}=\frac{{\tilde{L}}_{i}+{\tilde{L}}_{i+1}}{2}$. Additionally, $\tilde{l}=\left\{{\tilde{L}}_{1},...,{\tilde{L}}_{M}\right\}=\mathrm{sort}\left(l\right)$ where the sort operation (.) sorts a vector in increasing order. For the boundary cases, e.g., i=1,i=M, ${\tilde{L}}_{l,1}=L_{min}$, ${\tilde{L}}_{l,M}=L_{max}$, respectively.

### 5.2. Transmitter Identification Using Gaussian Mixture Modeling

The GMM consisted of Q=M+N component densities where only the Q=M densities could be trained. The 3Q GMM parameter was learned by running the expectation–maximization (EM) algorithm on the training data. The GMM, in its standard form, is perfectly suited for transmitter identification. Under the GMM, the probability density function (pdf) of the (observed) mixture random variable *X* is the convex/weighted sum of the component pdfs:

(14) $$f_{X}\left(x\right)=\sum_{q=1}^{Q}\pi_{q}\phi_{q}\left(x\right),$$

where each $\phi_{q}\left(x\right)$ is a Gaussian pdf that satisfies: $\phi_{q}\left(x\right)\geq 0$, $\int_{x\in R}\phi_{q}\left(x\right)dx=1$. The weights/priors satisfy: $\pi_{q}\left(x\right)\geq 0$, $\sum_{q=1}^{Q}\pi_{q}=1$.

The GMM has 3Q unknown parameters, which were learned by applying the iterative expectation–maximization algorithm on the training data ${\left\{x_{m}\right\}}_{m=1}^{M}$. The posterior probability for each point $x_{m}$ in the training data (i.e., the likelihood of $x_{m}$ belonging to component *q* of the mixture) was computed as follows (*j* is the iteration number):

(15) $$p_{m,q}^{\left(j\right)}=\frac{\pi_{q}^{\left(j\right)}\phi_{q}\left(x_{m},\mu_{q}^{\left(j\right)},\Sigma_{q}^{\left(j\right)}\right)}{\sum_{\hat{q}=1}^{Q}\pi_{\hat{q}}^{\left(j\right)}\phi \left(x_{m},\mu_{\hat{q}}^{\left(j\right)},\Sigma_{\hat{q}}^{\left(j\right)}\right)}.$$

The *Q* number of priors were updated as follows:

(16) $$\pi_{q}^{\left(j+1\right)}=\frac{1}{M}\sum_{m=1}^{M}p_{m,q}^{\left(j\right)}.$$

The *Q* number of means were updated as follows:

(17) $$\mu_{q}^{\left(j+1\right)}=\frac{\sum_{m=1}^{M}p_{m,q}^{\left(j\right)}x_{m}}{\sum_{m=1}^{M}p_{m,q}^{\left(j\right)}}.$$

The *Q* number of (co-)variances were updated as follows:

(18) $$\Sigma_{q}^{\left(j+1\right)}=\frac{\sum_{m=1}^{M}p_{m,q}^{\left(j\right)}\left(x_{m}-\mu_{q}^{\left(j\right)}\right){\left(x_{m}-\mu_{q}^{\left(j\right)}\right)}^{T}}{\sum_{m=1}^{M}p_{m,q}^{\left(j\right)}}.$$

The iterative EM algorithm monotonically increased the objective (likelihood) function value and converged when the increase in the likelihood function value between two successive iterations became less than the threshold ϵ.

**Figure 3:**
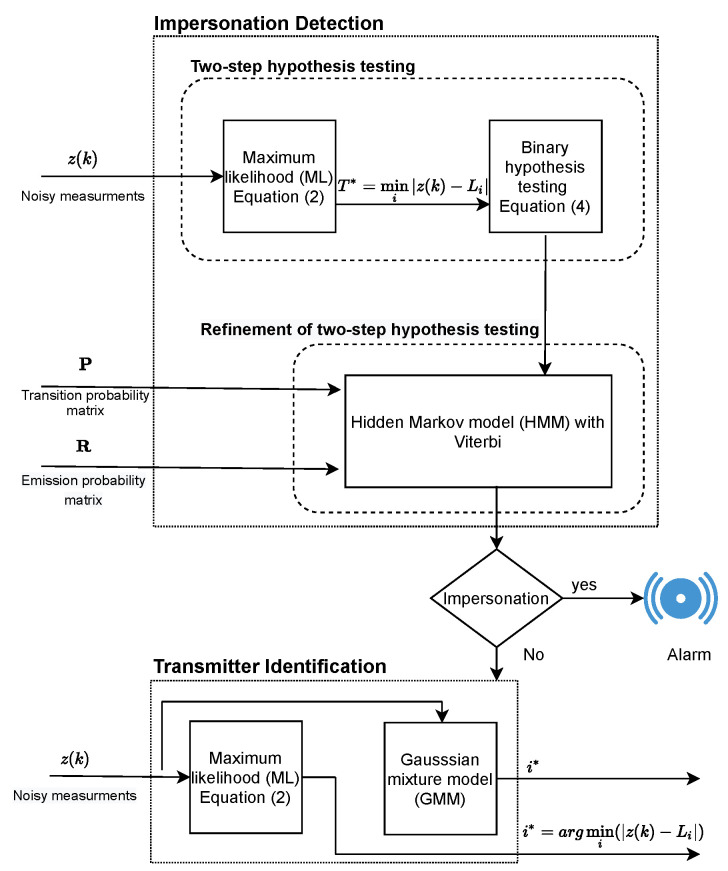
Proposed methodology for impersonation detection and transmitter identification in body-centric nanoscale communication systems operating in the THz band.

Figure 3 shows a flow graph of the proposed methodology. The noisy estimated measurement/path loss z(k) at slot *k* was fed to a two-step mechanism for impersonation detection, and the HMM was used to refine the outcomes of the two-step mechanism with the help of transition and emission probability matrices (i.e., P and R) and the Viterbi algorithm. Transmitter identification was done via the ML and GMM approaches when no impersonation was decided.

## 6. Simulations

### 6.1. Setup

We kept M=N=10, $\alpha_{ij}=0.5\;\forall j$, f=1 THz, T=285 k, and p=1 atm. Both the Alice and Eve nodes were deployed according to the uniform distribution in a 1 m × 1 m area. A total of $10^{5}$ random realizations (independent of the Alice and Eve nodes) of the nodes’ deployment were taken, and then, errors were averaged over the realizations.

$P_{fa}$ and $P_{md}$ are two well-known probabilities resulting in hypothesis testing. $P_{fa}$ was defined as the probability that any *i*-th Alice node can be considered as any of the Eve nodes $P_{md}$ is the probability of the event that any *j*-th Eve node can be considered as any of the Alice nodes.

### 6.2. Results

**Figure 4:**
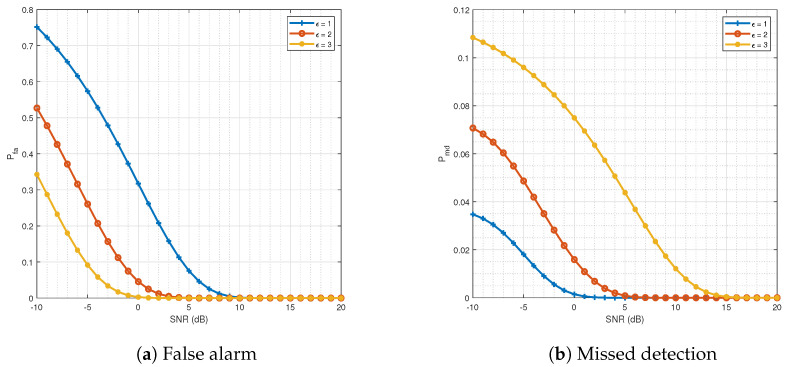
The error probabilities against SNR = $\frac{1}{\sigma^{2}}$ with different values for threshold ϵ. (**a**) Probability of false alarm. (**b**) Probability of missed detection. Both probabilities are decreasing functions of SNR.

Figure 4 represents the two probabilities against SNR $=\frac{1}{\sigma^{2}}$ where the improvement in error probabilities with an increasing SNR can be seen clearly. The designed parameter ϵ decreased $P_{md}$, but increased $P_{fa}$.

**Figure 5:**
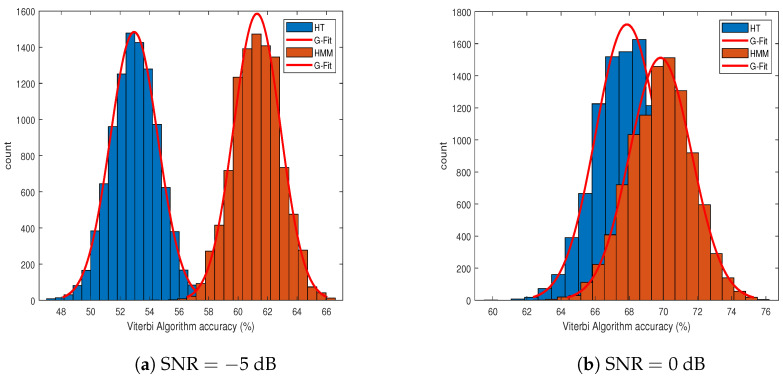
Performance comparison of two-step hypothesis testing and the hidden Markov model (HMM) with Viterbi algorithm. (**a**) Histogram comparison for a highly degraded channel, i.e., SNR = −5 dB. (**b**) Histogram comparison for a moderately degraded channel, i.e., SNR = 0 dB. Performances of both approaches get closer and closer when SNR increases.

**Figure 6:**
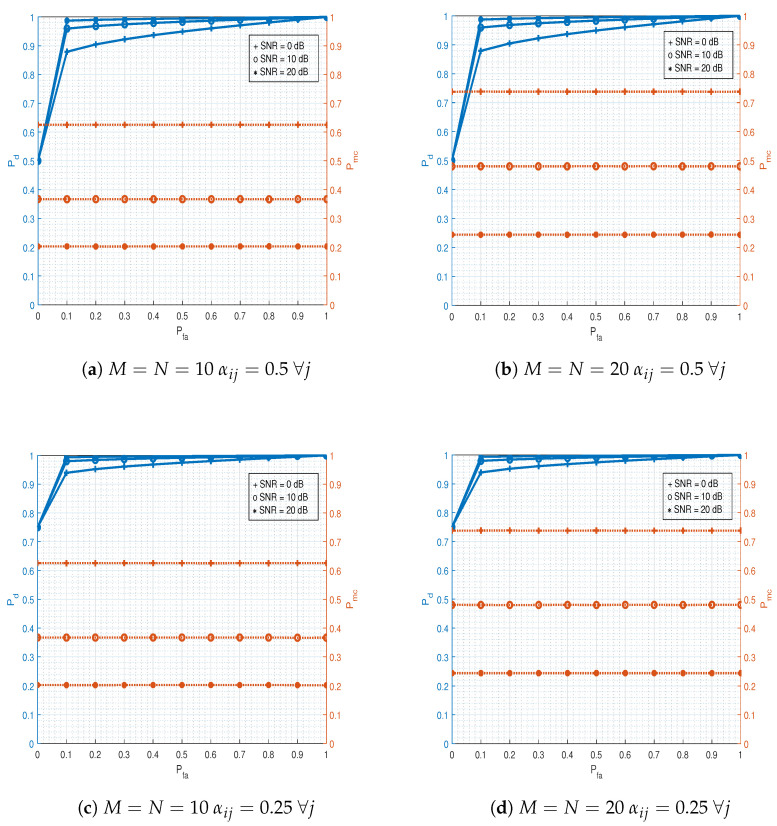
Receiver operating characteristic (ROC) curves: Three probabilities (false alarm, detection, and misclassification) are considered in the ROC. To study the impact of nodes, subfigures (**a**,**b**) are plotted. (**a**) Ten numbers of legitimate and malicious nodes are considered with 0.5 prior probability for a *j*-th malicious node. (**b**) Twenty numbers of legitimate and malicious nodes are considered with 0.5 prior probability for a *j*-th malicious node. Further, subfigures (**c**,**d**) are plotted to see the impact of transmissions /prior probabilities of malicious nodes. (**c**) Ten numbers of legitimate and malicious nodes are considered with 0.25 prior probability for a *j*-th malicious node. (**d**) Twenty numbers of legitimate and malicious nodes are considered with 0.25 prior probability for a *j*-th malicious node.

Figure 5 shows the efficacy of the HMM. At a low SNR, the performance of the HMM was far better than HT, and at a high SNR, HT was close to the HMM. The results were produced after the Monte Carlo-based simulation. The total number of transmissions was kept to $10^{5}$ (more specifically, $10^{5}$ binary states ($s_{0}$, $s_{1}$) were generated), ϵ=1, $P=0.5I_{2\times 2}$, where *I* is the identity matrix and $K=10^{3}$. The errors resulting from the HT and HMM methods were calculated as the number of times the predicted/estimated state was not equal to the actual state divided by the total transmissions. The accuracy was then computed accordingly. The entries of R were calculated according to $P_{fa}$ and $P_{md}$. Figure 6 shows the receiver operating characteristic (ROC) curves for different configurations of the nodes and transmissions from Eve nodes (i.e., $\alpha_{ij}$). Typically, the ROC contains two error probabilities ($P_{d}$ and $P_{fa}$), but due to multiple nodes in this study, we had three probabilities. For any $P_{fa}$ value, $P_{mc}$ was constant, which is obvious from Equation (13). Increasing the SNR not only improved $P_{d}$, but also improved $P_{mc}$ as well. $P_{fa}$ was chosen as an independent variable and swept in the range from zero to one. Using Equation (6), the threshold was calculated for a given SNR value. Further, $P_{d}=1-P_{md}$ (the detection probability) and $P_{mc}$ were computed as the average after doing $10^{5}$ uniform realizations of the nodes’ deployment. We observed that increasing the number of nodes did not affect $P_{d}$, but $P_{mc}$ increased with an increase in the number of Alice nodes (*M*). We further observed that when fewer nodes (Alice nodes) remained idle during their allocated slot, the more $P_{d}$ we had.

$P_{mc}$ is the probability of deciding the *i*-th Alice node, as any Alice node without *i*. $P_{mc}$ becomes an important metric when dealing with multiple nodes’ identification. Here, $P_{mc}$ resulted from both transmitter identification algorithms (ML, which is a bi-product of two-step HT-based authentication and the GMM). As the GMM is a learning approach, it requires training data to learn its parameters. That is the reason that we only performed transmitter identification using the GMM. We assumed no data were available for Eve nodes.

**Figure 7:**
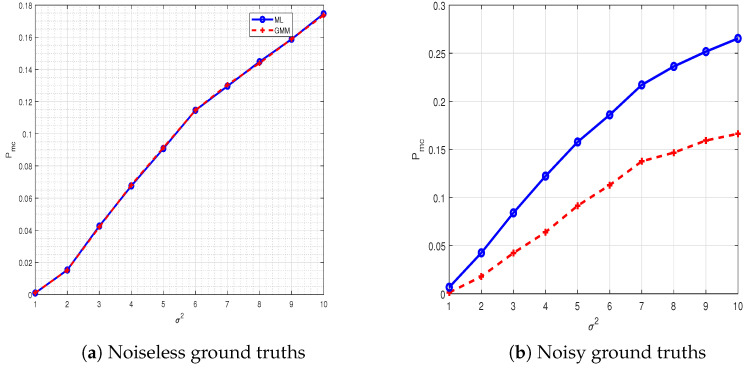
Misclassification error $P_{mc}$ against estimation error $\sigma^{2}$ for two-step hypothesis testing and GMM. (**a**) A scenario is considered where the perfect ground truth vector, i.e., l is obtained via a secure channel. (**b**) A scenario is considered where acquired ground truths are noisy. GMM has an advantage over ML when ground truths are noisy.

Figure 7a was generated by assuming actual ground truths (noiseless $\left(L_{i}\;\forall i\right)$) of Alice nodes available for performing ML-based transmitter identification. The ML was implemented using Equation (2) having noiseless ground truths. Figure 7a shows that the two approaches performed equally. To test the efficacy of the GMM approach, we performed another experiment and plotted the results in Figure 7b. This time, we assumed that the ground truths of the Alice nodes were noisy $L_{i}+n\;\forall i$ (i.e., when the ground truths were obtained on a secure channel, it also included noise or an error). This time, the ML-based approach was implemented using Equation (2) to include noisy ground truths. The GMM parameters were estimated on $10^{4}$ training data generated from the legitimate nodes and then tested on $10^{5}$. The error was calculated as the number of times the estimated state was not equal to the actual value divided by the total transmissions for both approaches and for both cases. We observed from Figure 7b that the overall performance of GMM was improved. The performance improved even further for lower SNR or higher $\sigma^{2}$.

### 6.3. Discussions

- From Figure 4 and Figure 6, we learned that the path loss could be exploited as a fingerprint to carry out authentication in body-centric nanoscale communication systems operating in the THz band. In other words, the proposed mechanisms can be used as a first line of defense against impersonation attacks.
- The results of the proposed two-step mechanism can be improved by using an additional approach (i.e., HMM). In particular, at a low SNR, the improvement was quite significant.
- The results in Figure 4 and Figure 6 indicate that, under the impersonation detection problem, it is not possible to minimize both Pmd and $P_{fa}$ at the same time because of their conflicting nature. In other words, one could minimize one error type only by compromising the other error type.
- GMM (Learning-based scheme) performed the same as our proposed two-step mechanism in transmitter identification. However, we learned that slightly complex nature of the GMM could produce improvements when the ground truths of legitimate nodes are noisy.

## 7. Conclusions

This paper provided an authentication mechanism using path loss as a fingerprint at the physical layer in body-centric nanoscale communication systems operating in the terahertz band. The work’s importance was advocated by illustrating envisioned smart healthcare application of body-centric nanoscale communication systems. The complex and quantum insecure crypto measures can be complemented using this approach, which is simple and quantum secure (i.e., no encryption or shared secret key is involved). This was observed from ROC curves after doing the Monte Carlo-based simulation for nodes’ deployment under a uniform distribution that with a 20% false rate, the detection probability was almost one when operating with SNR =10 dB. For simulation purpose, nodes were deployed in a 1 m × 1 m area under a uniform distribution, and air was considered as a medium among the nodes, while the path loss was calculated using the HITRAN database.

## Data Availability

The HITRAN database can be found here: https://hitran.org.

## Abbreviations

The following abbreviations are used in this manuscript:

THz	Terahertz
HMM	Hidden Markov model
GMM	Gaussian mixture model
SNR	Signal-to-noise ratio
IoT	Internet of things
DoS	Denial of service
PL	Physical layer
AKE	Asymmetric key encryption
RSS	Received signal strength
CIR	Channel impulse response
CFR	Channel frequency response
2D	Two dimensional
ML	Maximum likelihood
CCDF	Cumulative complementary distribution function
MLSE	Maximum likelihood sequence estimation
EM	Expectation maximization
ROC	Receiver operating characteristics
HITRAN	High-resolution transmission molecular absorption

## Appendix A

The spreading loss is given as:

(A1) $$L_{s}\left(f,d\right)\left[dB\right]=20\log_{10}\left(\frac{4\pi fd}{c}\right),$$

where *c* is the speed of light. The absorption loss is given as:

(A2) $$L_{a}\left(f,d\right)=\frac{1}{\tau (f,d)},$$

where τ represents the transmittance of the signal and is given by the Beer–Lambert law:

(A3) $$\begin{array}{c} \tau \left(f,d\right)=e^{-k(f)d}, \end{array}$$

where *k* is the medium absorption coefficient, given as:

(A4) $$k\left(f\right)=\sum_{i,g}k_{i,g}\left(f\right),$$

where:

(A5) $$k_{i,g}\left(f\right)=\frac{p}{p_{0}}\frac{T_{0}}{T}Q_{i,g},\sigma_{i,g}\left(f\right)$$

where *i* is the isotopologue (a molecule that differs in isotropic composition), *g* is gas, $p_{0}\left(T_{0}\right)$ is standard pressure (temperature), $\sigma_{i,g}\left(f\right)$ is the absorption cross-section, and $Q_{i,g}$ is the molecular density given by:

(A6) $$Q_{i,g}=\frac{n}{V}q_{i,g}N_{A}=\frac{p}{RT}q_{i,g}N_{A},$$

where *R* is the gas constant, $N_{A}$ is the Avogadro constant, and $q_{i,g}$ is the mixing ratio for *i* of *g*. The absorption cross-section can be expressed as:

(A7) $$\sigma_{i,g}=S_{i,g}G_{i,g}\left(f\right),$$

where the line intensity $S_{i,g}$ and line shape $G_{i,g}\left(f\right)$ parameters can be computed using data from the HITRAN database [19].
